# Author Correction: Matrix viscoelasticity promotes liver cancer progression in the pre-cirrhotic liver

**DOI:** 10.1038/s41586-025-09947-3

**Published:** 2025-11-27

**Authors:** Weiguo Fan, Kolade Adebowale, Lóránd Váncza, Yuan Li, Md Foysal Rabbi, Koshi Kunimoto, Dongning Chen, Gergely Mozes, David Kung-Chun Chiu, Yisi Li, Junyan Tao, Yi Wei, Nia Adeniji, Ryan L. Brunsing, Renumathy Dhanasekaran, Aatur Singhi, David Geller, Su Hao Lo, Louis Hodgson, Edgar G. Engleman, Gregory W. Charville, Vivek Charu, Satdarshan P. Monga, Taeyoon Kim, Rebecca G. Wells, Ovijit Chaudhuri, Natalie J. Török

**Affiliations:** 1 Gastroenterology and Hepatology, Stanford University, Stanford, CA USA; 2 VA, Palo Alto, CA USA; 3 Department of Chemical Engineering, Stanford University, Stanford, CA USA; 4 Chemistry, Engineering and Medicine for Human Health (ChEM-H), Stanford University, Stanford, CA USA; 5 Weldon School of Biomedical Engineering, Purdue University, West Lafayette, IN USA; 6 Department of Pathology, Stanford University, Stanford, CA USA; 7 Division of Immunology, Stanford University, Stanford, CA USA; 8 Department of Automation, Tsinghua University, Beijing, China; 9 Pittsburgh Liver Research Center, University of Pittsburgh and University of Pittsburgh Medical Center, Pittsburgh, PA USA; 10 Department of Radiology, Stanford University, Stanford, CA USA; 11 Department of Biochemistry and Molecular Medicine, University of California at Davis, Sacramento, CA USA; 12 Department of Molecular Pharmacology, Albert Einstein College of Medicine, New York, NY USA; 13 Quantitative Sciences Unit, Department of Medicine, Stanford University School of Medicine, Stanford, CA USA; 14 Faculty of Science and Technology, Keio University, Yokohama, Japan; 15 Departments of Medicine and Bioengineering, University of Pennsylvania, Philadelphia, PA USA; 16 Department of Mechanical Engineering, Stanford University, Stanford, CA USA

**Keywords:** Liver cancer, Non-alcoholic steatohepatitis, Biophysics

Correction to: *Nature* 10.1038/s41586-023-06991-9 Published online 31 January 2024

In the version of this article initially published, due to mistakes when outputting final source data, the plot shown in Fig. 1n and Fig. 1n source data were incorrect. The conclusions are unaffected by the change. The source data and figure panel are updated in the HTML and PDF versions of the article.

