# Supplementary material for: Matrix viscoelasticity promotes liver cancer progression in the pre-cirrhotic liver
Source: Nature. 2024 Jan 31;626(7999):635–42. doi: 10.1038/s41586-023-06991-9 (PMC10866704; doi:10.1038/s41586-023-06991-9)
Supplement: Supplementary file 1 — Supplementary Figs. 1 and 2, Supplementary Tables 1–5 and Supplementary Methods. [file 41586_2023_6991_MOESM1_ESM.pdf]

nature portfolio

https://doi.org/10.1038/s41586-023-06991-9

Supplementary information

Matrix viscoelasticity promotes liver cancer progression in the pre-cirrhotic liver

In the format provided by the authors and unedited

SUPPLEMENTARY INFORMATION

Matrix Viscoelasticity Promotes Liver Cancer Progression in the Pre-Cirrhotic Liver

Authors:

Weiguo Fan[1], Kolade Adebowale[2,3†], Lóránd Váncza[1†], , Yuan Li[1], Md Foysal Rabbi[4], Koshi Kunimoto[1], Dongning Chen[1], Gergely Mozes[1], David Kung-Chun Chiu[6, 7], Yisi Li[8], Junyan Tao[9], Yi Wei[1], Nia Adeniji[1], Ryan L. Brunsing[10], Renumathy Dhanasekaran[1], Aatur Singhi[9], David Geller[9], Su Hao Lo[11], Louis Hodgson[12], Edgar G. Engleman[6, 7], Gregory W. Charville[6], Vivek Charu[6,13], Satdarshan P. Monga[9], Taeyoon Kim [4, 5,] Rebecca G. Wells[14], Ovijit Chaudhuri[3,15] and Natalie J. Török[1*]

Table of contents:

Page 2: Supplementary Figure 1: Images of unprocessed blots used for the Extended Data Fig. 6g.

Page 2-3: Supplementary Figure 2: Images of unprocessed blots used for the Extended Data Fig. 11a.

Page 4: Supplementary Table1: Patient information

Page 5: Supplementary Table2: List of primer sequences for the CRISPR/Cas9 studies.

Page 6-7: Supplementary Table3: List of antibodies used for the various experiments.

Page 8: Supplementary Table 4. List of primer sequences for RTqPCR.

Page 9: Supplementary Table 5. List of parameters employed in the computational model.

Page 10-14: Supplementary Methods

Supplementary Figure 1: Images of unprocessed blots used for the Extended data Fig. 6g

Samples were run on the different gels.

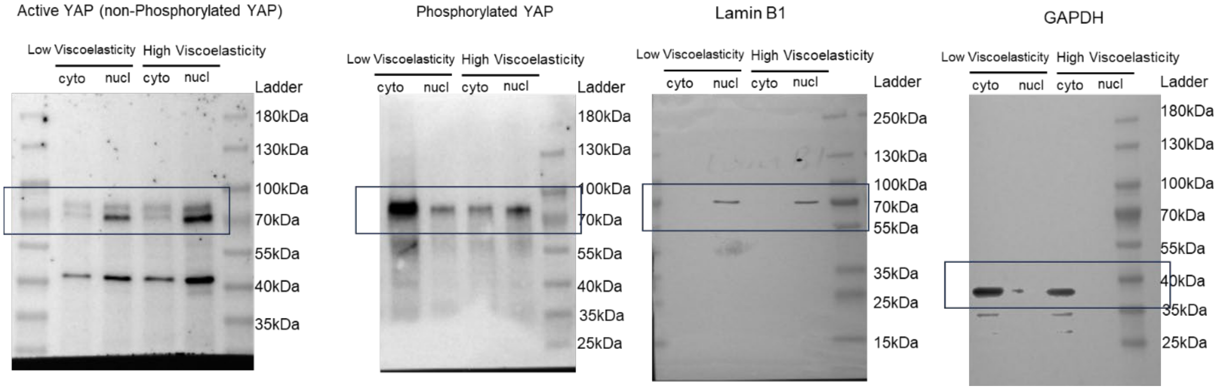

Supplementary Figure 2: Images of unprocessed blots used for the Extended data Fig. 11a

*S*amples were run on the different gels.

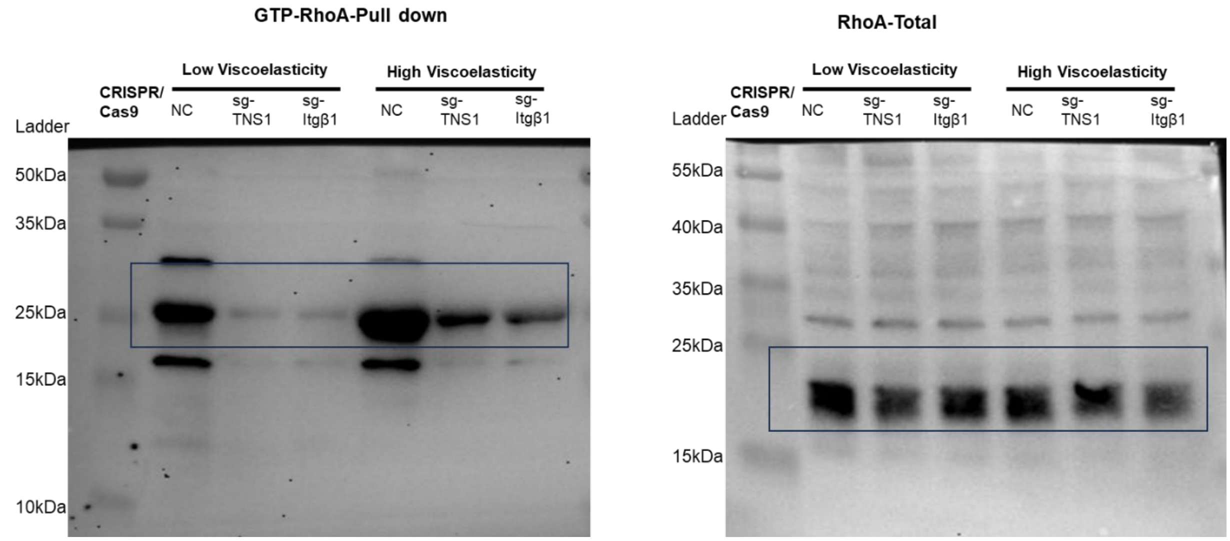

p-LATS1

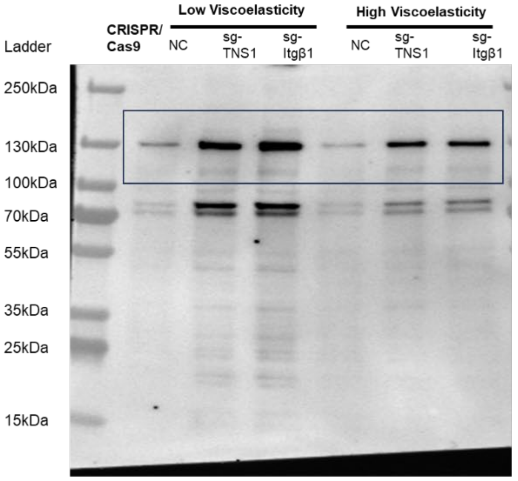

LATS1

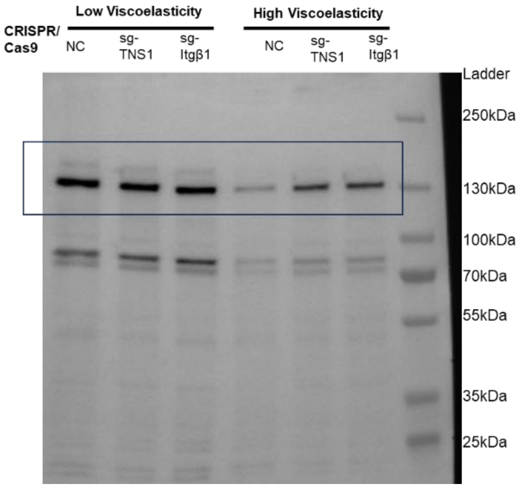

Active YAP (non-Phosphorylated YAP)

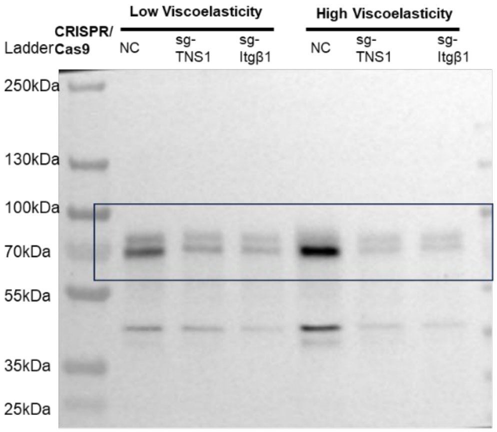

Phosphorylated YAP

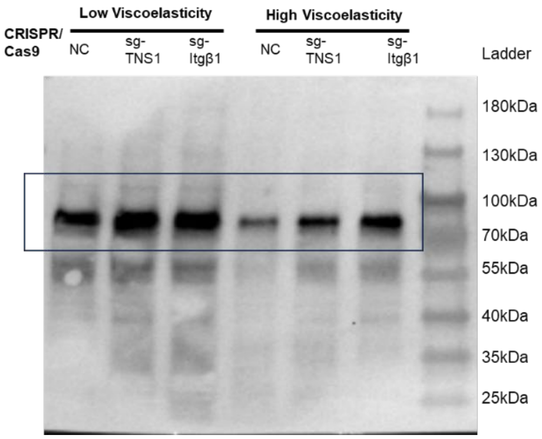

GAPDH

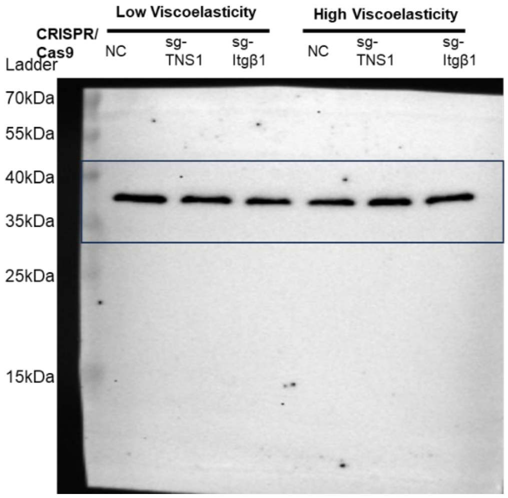

Table 1. Patient information

Serial no.	Age	Sex	Clinical Diagnosis	NAS Score
1	61	Female	MASH	1
2	56	Male	Healthy	0
3	40	Male	MASH/Diabetes	6
4	58	Female	Diabetes	0
5	51	Male	MASH	6
6	38	Female	MASH/Diabetes	2
7	50	Male	Healthy	0
8	70	Male	Diabetes	0
9	47	Female	Healthy	0
10	52	Female	MASH	4
11	66	Male	MASH	3
12	58	Male	MASH	3
13	66	Male	MASH/Diabetes	4
14	59	Female	MASH/Diabetes	5
15	81	Female	MASH	3
16	47	Female	Healthy	0
17	75	Female	Diabetes	0
18	57	Male	Diabetes	0
19	37	Male	MASH/Diabetes	4
20	59	Male	MASH/Diabetes	4

Table 2. List of primer sequences for the CRISPR/Cas9 studies.

gene	Primer Sequence
**Mouse *TNS1***	sgRNA1: 5’- GAGCTGGACCGCCTACTAAGTGG-3’<br><br>sgRNA2: 5’- AAGTTGACGATGGCAGACGCAGG-3’
**Human *TNS1***	sgRNA1, 5’- GTAGAACAACGACATTGTGA-3’<br><br>sgRNA2, 5’- TGGCTACAAGACTCTCCAAG-3’<br><br>sgRNA3, 5’-CCCAACTTTGAGTCTAAAGG -3’
**Human *Integrin β1***	sgRNA1, 5’- TTGGCTGGAGGAATGTTACA-3’<br><br>sgRNA2, 5’-GAACGGGGTGAATGGAACAG-3’<br><br>sgRNA3, 5’-TAGGCCTCTGGGCTTTACGG-3’

Table 3. List of antibodies used for various experiments

**Primary Antibodies**	**Manufacturer/ Cat #**	**Application/ Concentration**
c-Myc	Santa Cruz , #sc-40	IHC, 1:200
Glutathione Synthase (GS)	Santa Cruz, #sc-74430	IHC, 1:200
Tensin 1 (TNS1)	Sigma-Aldrich, #SAB4200283	IF, 1:200; PLA, 1:100
Integrin β1 blocking (Itgb1)	Abcam, #ab24693	Cell culture, 1:100
Active Integrin β1 (12G10)	Abcam, # ab30394	IF, 1:200; PLA, 1:100
Active (non-phosphorylated) Yap	Abcam, # ab 205270	IF, 1:200; WB, 1:1000
MT1-MMP (MMP14)	Abcam, # ab 51074	IF, 1:200
Phospho-Myosin Light Chain 2 (Ser19)	Cell Signaling Technology, # 95777	IF, 1:200
phosphorylated Yap (Ser127)	Cell Signaling Technology, #4911	WB, 1:1000
GAPDH	Santa Cruz, # sc-365062	WB, 1:10,00
LATS1	Cell Signaling Technology, # 3477	WB, 1:10,00
Phospho-LATS1 (Thr1079)	Cell Signaling Technology, # 8654	WB, 1:10,00
**Secondary Antibodies**		
HRP Goat α-Rabbit	Abcam, #ab6721	WB, 1:5000
Alexa Fluor 488 Chicken α-Rabbit	Invitrogen, #A21441	IF, 1:500
Alexa Fluor 555 Goat α-Mouse	Invitrogen, #A21422	IF, 1:500

Alexa Fluor 555 Donkey α-Rabbit	Invitrogen, #A31572	IF, 1:500
Biotinylated Goat α-Mouse	Vector Lab,#BA-9200	IHC, 1:500

Table 4. List of primer sequences for RTqPCR.

mRNA	Primer Sequence
**Human *GAPDH***	Forward: 5'- GAAATCCCATCACCATCTTCCAGG-3'<br>Reverse: 5'-GAGCCCCAGCCTTCTCCATG -3'
**Human *CTGF***	Forward: 5'- GACGAGCCCAAGGACCAAAC -3'<br>Reverse: 5'- TCATAGTTGGGTCTGGGCCA-3'
**Human Cyr61**	Forward: 5'-AGCCTCGCATCCTATACAACC -3'<br>Reverse: 5'-TTCTTTCACAAGGCGGCACTC-3'
**Human *TNS1***	Forward: 5'- GTACGTCACAGAGAGGATCATCG -3'<br>Reverse: 5'-GCAGGTAGTTGCCTCCATGTT -3'
**Mouse *Arbp***	Forward: 5'- CAAAGCTGAAGCAAAGGAAGAG -3'<br>Reverse: 5'-AATTAAGCAGGCTGACTTGGTTG-3'
**Mouse *TNS1***	Forward: 5'-AGAGACCGTACCCAAGAATGT -3'<br>Reverse: 5'- GTAGGCTGTGATTGTGGTTGT-3'
**Mouse *CTGF***	Forward: 5'-CACCTAAAATCGCCAAGCCTG-3'<br>Reverse: 5'-AGTTCGTGTCCCTTACTTCCTG-3'
**Mouse *Cyr61***	Forward: 5'-ACCGCTCTGAAAGGGATCTG-3'<br>Reverse: 5'-TGTTTACAGTTGGGCTGGAAG-3'

Table 5. List of parameters employed in the computational model.

Symbol	Definition	Value
$r_{0,\mathrm{f}}$	Equilibrium length of fibril segments	5.0×10^{-7} [m]
$r_{\mathrm{c,f}}$	Diameter of fibril segments	1.0×10^{-8} [m]
$\theta_{0,\mathrm{f}}$	Bending angle formed by adjacent fibril segments	0 [rad]
$\kappa_{\mathrm{s,f}}$	Extensional stiffness of fibrils	1.59×10^{-2} [N/m]
$\kappa_{\mathrm{b,f}}$	Bending stiffness of fibrils	1.67×10^{-19} [N·m]
$r_{0,\mathrm{bu}}$	Equilibrium length of a bundler arm	2.0×10^{-8} [m]
$r_{\mathrm{c,bu}}$	Diameter of a bundler arm	2.0×10^{-8} [m]
$\theta_{0,\mathrm{bu},1}$	Bending angle formed by two bundler arms	0 [rad]
$\theta_{0,\mathrm{bu},2}$	Bending angle formed by mother and daughter fibrils	0-30 [deg]
$\kappa_{\mathrm{s,bu}}$	Extensional stiffness of cross-linkers	2.0×10^{-3} [N/m]
$\kappa_{\mathrm{b,bu},1}$	Bending stiffness 1 of cross-linkers	1.04×10^{-18} [N·m]
$\kappa_{\mathrm{b,bu},2}$	Bending stiffness 2 of cross-linkers	1.04×10^{-18} [N·m]
$r_{0,\mathrm{xl}}$	Equilibrium length of a cross-linker arm	2.0×10^{-8} [m]
$r_{\mathrm{c,xl}}$	Diameter of a cross-linker arm	2.0×10^{-8} [m]
$\theta_{0,\mathrm{xl}}$	Bending angle formed by two cross-linker arms	0 [rad]
$\kappa_{\mathrm{s,xl}}$	Extensional stiffness of cross-linkers	2.0×10^{-3} [N/m]
$\kappa_{\mathrm{b,xl}}$	Bending stiffness of cross-linkers	1.04×10^{-17} [N·m]
κ_{r}	Strength of repulsive force	1.69×10^{-3} [N/m]
$k_{+,\mathrm{xl}}$	Binding rate of cross-linkers	1×10^{2} [μM^{-1}s^{-1}]
$k^{0}_{-,\mathrm{xl}}$	Zero-force unbinding rate constant of cross-linkers	1×10^{-6} [s^{-1}]
$x_{-,\mathrm{xl}}$	Force sensitivity of cross-linker unbinding	4.0×10^{-10} [m]
$<L_{\mathrm{f}}>$	Average length of fibers	3 or 5 [μm]
C_{f}	Fiber density	2.42 or 1.54 [fibril/μm^{3}]
C_{bu}	Bundler density	1.68 [bundler/μm^{3}]
C_{xl}	Cross-linking density	120 [cross-linker/μm^{3}]
γ	Shear strain	0.2 (= 20 %)
Δt	Time step	4.91×10^{-5} [s]
μ	Viscosity of medium	0.86 [Pa·s]
$k_{\mathrm{B}}T$	Thermal energy	4.142×10^{-21} [J]

Supplementary Methods for Simulation Modeling

Brownian dynamics based on the Langevin equation

The displacements of the cylindrical elements at each step are governed by the Langevin equation with negligence of inertia:

$$\mathbf{F}_i - \zeta_i \frac{\mathrm{d}\mathbf{r}_i}{\mathrm{d}t} + \mathbf{F}_i^{\mathrm{T}} = 0 \tag{S1}$$

where $\mathbf{r}_i$ indicates the position of the ith element, ζ_i is a drag coefficient, $\mathbf{F}_i$ is a deterministic force, and t is time. The stochastic force $\mathbf{F}^{\mathrm{T}}_i$ satisfies the fluctuation-dissipation theorem [1]:

$$\left\langle \mathbf{F}_i^{\mathrm{T}}(t)\mathbf{F}_j^{\mathrm{T}}(t)\right\rangle = \frac{2k_{\mathbf{B}}T\zeta_i\delta_{ij}}{\Delta t}\boldsymbol{\delta} \tag{S2}$$

where δ_{ij} is the Kronecker delta, $\boldsymbol{\delta}$ is a second-order tensor, and Δt is a time step.

The drag coefficients are calculated using an approximated form for a cylindrical object [2]:

$$\zeta_i = 3\pi\mu r_{\mathrm{c},i}\frac{3+2r_{0,i}/r_{\mathrm{c},i}}{5} \tag{S3}$$

where μ is the viscosity of a surrounding medium, and $r_{0,i}$ and $r_{\mathrm{c},i}$ are the length and diameter of an element, respectively. To update the positions of all cylindrical elements at each time step, we use the Euler integration scheme:

$$\mathbf{r}_i(t+\Delta t) = \mathbf{r}_i(t) + \frac{\mathrm{d}\mathbf{r}_i}{\mathrm{d}t}\Delta t = \mathbf{r}_i(t) + \frac{1}{\zeta_i}\left(\mathbf{F}_i + \mathbf{F}_i^{\mathrm{T}}\right)\Delta t \tag{S4}$$

Deterministic forces

Deterministic forces include extensional and bending forces that maintain equilibrium lengths and equilibrium angles as well as repulsive forces accounting for volume-exclusion effects

between cylindrical elements that represent fibrils. Extensional and bending forces for fibrils, bundlers, and cross-linkers originate from harmonic potentials:

$$U_{\mathrm{s}} = \frac{1}{2}\kappa_{\mathrm{s}}\left(r - r_0\right)^2 \tag{S5}$$

$$U_{\mathrm{b}} = \frac{1}{2}\kappa_{\mathrm{b}}\left(\theta - \theta_0\right)^2 \tag{S6}$$

where κ_{s} and κ_{b} are extensional and bending stiffnesses, r and r_0 are the instantaneous and equilibrium lengths of cylindrical elements, and θ and θ_0 are instantaneous and equilibrium angles formed by interconnected elements.

Simplified geometry and mechanics of matrix elements

Fibrils are comprised of serially connected cylindrical segments. The equilibrium length of fibril elements ($r_{0,\mathrm{f}}$= 0.5 μm) and an equilibrium angle formed by two adjacent fibril elements ($\theta_{0,\mathrm{f}}$ = 0 rad) are maintained by extensional ($\kappa_{\mathrm{s,f}}$) and bending stiffnesses ($\kappa_{\mathrm{b,f}}$) of fibrils, respectively. The value of $\kappa_{\mathrm{b,f}}$ used in the model corresponds to the persistence length of ~20 μm. Bundlers consist of two cylindrical arm segments. The equilibrium length of a bundler arm ($r_{0,\mathrm{bu}}$ = 20 nm) is maintained by extensional stiffness ($\kappa_{\mathrm{s,bu}}$). An equilibrium angle between two arms of each bundler ($\theta_{0,\mathrm{bu},1}$ = 0 rad) is maintained by bending stiffness ($\kappa_{\mathrm{b,bu},1}$). An equilibrium angle between two fibrils connected by bundlers ($\theta_{0,\mathrm{bu},2}$) is maintained by additional bending stiffness ($\kappa_{\mathrm{b,bu},2}$). Cross-linkers also consist of two cylindrical arm segments. The equilibrium length of a cross-linker arm ($r_{0,\mathrm{xl}}$ = 20 nm) is maintained by extensional stiffness ($\kappa_{\mathrm{s,xl}}$), and an equilibrium angle formed by two arms of each cross-linker ($\theta_{0,\mathrm{xl}}$ = 0 rad) is maintained by bending stiffness ($\kappa_{\mathrm{b,xl}}$). Forces exerted on fibril elements by bundlers or cross-linkers are distributed onto two nodes located at the ends of the fibril element.

Repulsive forces between fibrils prevent the fibrils from passing through each other. A minimum distance between two cylindrical elements, r_{r}, is computed, and the repulsive force originates from the following harmonic potential ($U_{\mathrm{r,f}}$):

$$U_{\mathrm{r,f}} = \begin{cases} \frac{1}{2}\kappa_{\mathrm{r,f}}\left(r_{\mathrm{r}} - r_{0,\mathrm{r}}\right)^2 & \text{if} \quad r_{\mathrm{r}} < r_{0,\mathrm{r}} \\ 0 & \text{if} \quad r_{\mathrm{r}} \geq r_{0,\mathrm{r}} \end{cases} \tag{S7}$$

where $\kappa_{\mathrm{r,f}}$ is the strength of repulsive force, and $r_{0,\mathrm{r}}$ is a critical distance.

Dynamics of fibrils, bundlers, and cross-linkers

Nucleation of seed fibrils takes place via appearance of one cylindrical segment in random positions. It is quickly elongated in one direction up to either 3 μm or 5 μm via addition of segments. Depolymerization of fibrils is not considered.

Arms of bundlers irreversibly bind to binding sites located every 50 nm on fibril elements. One of the bundler arms rapidly binds to the end or mid of fibrils at a rate, $k_{+,\mathrm{xl}}$. The part of the mother fibril where the bundler arms can bind is specified by two boundaries, b_1 and b_2, between 0 and 1 (**Extended Data Fig. 5c).** Then, a daughter fibril is instantaneously nucleated on the other arm via appearance of one segment with a given angle relative to the mother fibril, followed by elongation of the daughter fibril up to 3 μm or 5 μm. The length of bundles is controlled by varying b_1 and b_2. For example, longer bundles are formed if a bundler can bind to one end of mother fibrils (i.e., large b_1 and b_2). By contrast, shorter bundles are formed if a bundler can bind only to either the other end of mother fibrils ($b_1 = b_2 = 0$) or part close to the other end (i.e., small b_1 and b_2). The angle between mother and daughter fibrils determines a bundle shape. If the angle is zero,

fibrils in bundles are parallel to each other, leading to formation of tight bundles, whereas larger angles result in loose bundles with splaying fibrils.

Arms of cross-linkers also bind to binding sites located every 50 nm on fibril elements at a rate, $k_{+,\mathrm{xl}}$, without preference of contact angle. Cross-linker arms also unbind from fibers at a force-dependent rate, $k_{-,\mathrm{xl}}$, determined by Bell's law [45]:

$$k_{-,\mathrm{xl}} = \begin{cases} k^{0}_{-,\mathrm{xl}} \exp\left(\dfrac{F_{\mathrm{s,xl}} x_{-,\mathrm{xl}}}{k_{\mathrm{B}}T}\right) & \text{if } r \geq r_{0,\mathrm{xl}} \\ k^{0}_{-,\mathrm{xl}} & \text{if } r < r_{0,\mathrm{xl}} \end{cases} \tag{S8}$$

Where $k^{0}_{-,\mathrm{xl}}$ is a zero-force unbinding rate constant, $x_{-,\mathrm{xl}}$ represents sensitivity to the magnitude of an applied spring force ($F_{\mathrm{s,xl}}$), $r_{0,\mathrm{xl}}$ is the equilibrium length of a cross-linker element, and $k_{\mathrm{B}}T$ is thermal energy. The values of $k^{0}_{-,\mathrm{xl}}$ (= 10^{-6} s^{-1}) and $x_{-,\mathrm{xl}}$ (= 4.0×10^{-10} m) are determined by benchmarking *in-vitro* experimental results as shown in our previous study [46].

Matrix assembly and bulk rheology

The computational domain is rectangular with 20×20×5 μm in x, y, and z directions. A periodic boundary condition exists only in x and z directions. First, fibrils are assembled with or without bundlers. Some of the fibrils are permanently bound to two boundaries normal to the y direction (i.e., +y boundary located at y = 20 μm and – y boundary located at y = 0 μm) during the formation of individual fibrils or bundles. After completion of fibril/bundle assembly, a large number of cross-linkers are added to connect fibrils or bundles into a matrix. Then, without cross-linker unbinding, 20% shear strain is applied to the +y boundary in the +x direction to mimic bulk rheology, whereas the -y boundary is fixed. After reaching the 20% strain, the strain is held at a constant level for 10 s without cross-linker unbinding to relax initial excessive stress originating

from drag forces. Then, cross-linker unbinding is activated to measure stress relaxation. Stress at each time point is calculated by summing all forces acting on the +y boundary in the x direction and then dividing the sum by the area of the boundary.
