# Supplementary material for: Matrix viscoelasticity promotes liver cancer progression in the pre-cirrhotic liver
Source: Nature. 2024 Jan 31;626(7999):635–42. doi: 10.1038/s41586-023-06991-9 (PMC10866704; doi:10.1038/s41586-023-06991-9)
Supplement: Supplementary file 2 — Reporting Summary [file 41586_2023_6991_MOESM2_ESM.pdf]

nature portfolio

Corresponding author(s): Natalie Torok

Last updated by author(s): Oct 18, 2023

Reporting Summary

Nature Portfolio wishes to improve the reproducibility of the work that we publish. This form provides structure for consistency and transparency in reporting. For further information on Nature Portfolio policies, see our Editorial Policies and the Editorial Policy Checklist.

Statistics

For all statistical analyses, confirm that the following items are present in the figure legend, table legend, main text, or Methods section.

n/a	Confirmed	
☐	☒	The exact sample size (*n*) for each experimental group/condition, given as a discrete number and unit of measurement
☐	☒	A statement on whether measurements were taken from distinct samples or whether the same sample was measured repeatedly
☐	☒	The statistical test(s) used AND whether they are one- or two-sided<br>*Only common tests should be described solely by name; describe more complex techniques in the Methods section.*
☐	☒	A description of all covariates tested
☐	☒	A description of any assumptions or corrections, such as tests of normality and adjustment for multiple comparisons
☐	☒	A full description of the statistical parameters including central tendency (e.g. means) or other basic estimates (e.g. regression coefficient) AND variation (e.g. standard deviation) or associated estimates of uncertainty (e.g. confidence intervals)
☐	☒	For null hypothesis testing, the test statistic (e.g. *F*, *t*, *r*) with confidence intervals, effect sizes, degrees of freedom and *P* value noted<br>*Give P values as exact values whenever suitable.*
☒	☐	For Bayesian analysis, information on the choice of priors and Markov chain Monte Carlo settings
☒	☐	For hierarchical and complex designs, identification of the appropriate level for tests and full reporting of outcomes
☒	☐	Estimates of effect sizes (e.g. Cohen's *d*, Pearson's *r*), indicating how they were calculated

Our web collection on statistics for biologists contains articles on many of the points above.

Software and code

Policy information about availability of computer code

Data collection	Histology and Immunohistochemistry Images were scanned with Leica Aperio AT2 System.<br>High resolution IFC images were obtained by the ZEISS Airyscan2 LSM980 microscope.<br>AFM Data analyses were performed using the Hertz model in NanoScope Analysis V1.9, and Mountains SPIP v.9.<br>Rheometry was carried out on an ARES-G2 rheometer (TA instruments) by using TA TRIOS software V5.1.1 (TA instruments).<br>Second Harmonic Generation images and fluorescent images were obtained using Leica TCS SP8, multi-photon Leica Stellaris 8 DIVE upright Confocal, and ZEISS Airyscan2 LSM980.<br>Immunoblotting images were collected using the Invitrogen iBright Imaging Systems (Thermo Fisher Scientific - US).<br>RNA-seq data were collected on an Illumina NovaSeq 6000 Sequencing with paired-end 150bp reads.
Data analysis	The AFM data and Second Harmonic Generation images were analyzed using Mountains SPIP v.9, CT Fire software (V2.0 beta), and NIH Image J software (Version 1.53t).<br>Immunoblotting images were analyzed using iBright Analysis Software (Version 5.2.1).<br>Statistical analyses were performed using Graphpad Prism (GraphPad Software, version 10).<br>For RNA-seq analysis,Gencode gene annotations version M18, GRCm38 (https://www.gencodegenes.org/), Dropseq tools v1.1249, R v3.4.4., Limma v3.40.650 were used.<br>GSEA was conducted with the pre-ranked GSEA method within the KEGG databases with the online tool g:Profiler (https://biit.cs.ut.ee/gprofiler/gost). RNA-seq Heatmaps and unsupervised hierarchical clustering was performed with g:Profiler (https://biit.cs.ut.ee/gprofiler/gost).

For manuscripts utilizing custom algorithms or software that are central to the research but not yet described in published literature, software must be made available to editors and reviewers. We strongly encourage code deposition in a community repository (e.g. GitHub). See the Nature Portfolio guidelines for submitting code & software for further information.

Data

Policy information about availability of data

All manuscripts must include a data availability statement. This statement should provide the following information, where applicable:

- Accession codes, unique identifiers, or web links for publicly available datasets
- A description of any restrictions on data availability
- For clinical datasets or third party data, please ensure that the statement adheres to our policy

We do not have any restrictions on data availability. All data generated during this study, are included in the article and the supplementary information files. The mouse reference genome major release GRCm38 are available from https://www.gencodegenes.org/.
RNA-seq data are available under the accession number GSE245016. Codes used in the study are available at https://github.com/ktyman2/liverCancer.git

Human research participants

Policy information about studies involving human research participants and Sex and Gender in Research.

Reporting on sex and gender	We have obtained de-identified liver samples and studied them from 9 female and 11 male adult patients. Acquisition was according to the BRISQ Guidelines, and liver resection specimens that were stored at -80C or available fresh (for rheometry analyses) were studied.
Population characteristics	MASH patients (age between 30 to 80) with no HBV/HCV/HIV infection and no history of heavy alcohol drinking. Histology was evaluated for necroinflammation, hepatocellular ballooning, ductular reaction, and fibrosis by a hepato-pathologist in a blinded fashion, and NAS scores were provided.
Recruitment	Human liver samples were obtained from Stanford Diabetes Research Center (SDRC), Donor Network West (DNW), Stanford Tissue Bank, and the Clinical Biospecimen Repository and Processing Core (CBRPC) of the Pittsburgh Liver Research Center (PLRC). No identifying information was available.
Ethics oversight	All human samples were de-identified and exempted (Exemption 4). This was approved by Stanford University Institutional Review Board (IRB, #67378) and Pittsburgh Liver Research Center Review Board.

Note that full information on the approval of the study protocol must also be provided in the manuscript.

Field-specific reporting

Please select the one below that is the best fit for your research. If you are not sure, read the appropriate sections before making your selection.

☒ Life sciences ☐ Behavioural & social sciences ☐ Ecological, evolutionary & environmental sciences

For a reference copy of the document with all sections, see nature.com/documents/nr-reporting-summary-flat.pdf

Life sciences study design

All studies must disclose on these points even when the disclosure is negative.

Sample size	Sample size was determined by pilot experiments and resource availability.
Data exclusions	We have not performed any data exclusion.
Replication	All findings have been replicated with more than 3 biological repeats, and methods. The key findings were verified independently by other individuals.
Randomization	Mice in each independent experiment, as well as the experimental groups were age-matched. Mice were randomly distributed into each group ensuring similar age and body weight in each group. For in vitro experiments, cells and hydrogels were grouped randomly for treatment or test.
Blinding	Blinding was widely used in the study. The images were scored by at least two individuals who were blinded for the group information. Measurements of transformed foci were confirmed by a second person who was also blinded for the information on the experimental group. Investigators were blinded to allocation during experiments and outcome assessments, and data was collected and analyzed in a blinded fashion.

March 2021

Reporting for specific materials, systems and methods

We require information from authors about some types of materials, experimental systems and methods used in many studies. Here, indicate whether each material, system or method listed is relevant to your study. If you are not sure if a list item applies to your research, read the appropriate section before selecting a response.

Materials & experimental systems

n/a	Involved in the study	
☐	☒	Antibodies
☐	☒	Eukaryotic cell lines
☒	☐	Palaeontology and archaeology
☐	☒	Animals and other organisms
☒	☐	Clinical data
☒	☐	Dual use research of concern

Methods

n/a	Involved in the study	
☒	☐	ChIP-seq
☒	☐	Flow cytometry
☒	☐	MRI-based neuroimaging

Antibodies

Antibodies used

Myc Santa Cruz , #sc-40 IHC, 1:200
Glutamine Synthetase (GS) Santa Cruz, #sc-74430 IHC, 1:200
Tensin 1 (TNS1) Sigma-Aldrich, #SAB4200283 IF, 1:200; PLA, 1:100
Integrin β1 blocking (Itgb1) Abcam, #ab24693 Cell culture, 1:100
Active Integrin β1 (12G10) Abcam, # ab30394 IF, 1:200; PLA, 1:100
Active (non-phosphorylated) Yap Abcam, # ab 205270 IF, 1:200; WB, 1:1000
MT1-MMP (MMP14) Abcam, # ab 51074 IF, 1:200
Phospho-Myosin Light Chain 2 (Ser19) Cell Signaling Technology, # 95777 IF, 1:200
phosphorylated Yap (Ser127) Cell Signaling Technology, #4911 WB, 1:1000
GAPDH Santa Cruz, # sc-365062 WB, 1:10,00
LATS1 Cell Signaling Technology, # 3477 WB, 1:10,00
Phospho-LATS1 (Thr1079) Cell Signaling Technology, # 8654 WB, 1:10,00

Secondary Antibodies
HRP Goat α-Rabbit Abcam, #ab6721 WB, 1:5000
Alexa Fluor 488 Chicken α-Rabbit Invitrogen, #A21441 IF, 1:500
Alexa Fluor 555 Goat α-Mouse Invitrogen, #A21422 IF, 1:500
Alexa Fluor 555 Donkey α-Rabbit Invitrogen, #A31572 IF, 1:500
Biotinylated Goat α-Mouse Vector Lab,#BA-9200 IHC, 1:500

Validation

Myc https://www.scbt.com/p/c-myc-antibody-9e10
Glutamine Synthetase (GS) https://www.scbt.com/p/gl-syn-antibody-e-4
Tensin 1 (TNS1) https://www.sigmaaldrich.com/US/en/product/sigma/sab4200283
Integrin β1 blocking (Itgb1) https://www.abcam.com/integrin-beta-1-antibody-p5d2-ab24693.html
Active Integrin β1 (12G10) https://www.abcam.com/products/primary-antibodies/integrin-beta-1-antibody-12g10-ab30394.html
Active (non-phosphorylated) Yap https://www.abcam.com/products/primary-antibodies/active-yap1-antibody-epr19812-ab205270.html
MT1-MMP (MMP14) https://www.abcam.com/products/primary-antibodies/mmp14-antibody-ep1264y-ab51074.html
Phospho-Myosin Light Chain 2 (Ser19) https://www.cellsignal.com/products/primary-antibodies/phospho-myosin-light-chain-2-thr18-ser19-e2j8f-rabbit-mab/95777
phosphorylated Yap (Ser127) https://www.cellsignal.com/products/primary-antibodies/phospho-yap-ser127-antibody/4911?site-search-type=Products&N=4294956287&Ntt=%234911&fromPage=plp&_requestid=538663
GAPDH https://www.scbt.com/p/gapdh-antibody-g-9?gclid=Cj0KCQjwnf-kBhCnARIsAFlg490L7314d4A25tXUFjzFpS2TOuYoCeZvaUwYZ_Qixt_PgNhvTkFXH_AaAhL5EALw_wcB
LATS1 https://www.cellsignal.com/products/primary-antibodies/lats1-c66b5-rabbit-mab/3477?site-search-type=Products&N=4294956287&Ntt=%23+3477+&fromPage=plp&_requestid=538731
Phospho-LATS1 (Thr1079) https://www.cellsignal.com/products/primary-antibodies/phospho-lats1-thr1079-d57d3-rabbit-mab/8654?site-search-type=Products&N=4294956287&Ntt=8654+&fromPage=plp&_requestid=538760
Secondary Antibodies:
HRP Goat α-Rabbit Abcam, #ab6721 https://www.abcam.com/products/secondary-antibodies/goat-rabbit-igg-hl-hrp-ab6721.html
Alexa Fluor 488 Chicken α-Rabbit Invitrogen, #A21441https://www.thermofisher.com/antibody/product/Chicken-anti-Rabbit-IgG-H-L-Cross-Adsorbed-Secondary-Antibody-Polyclonal/A-21441
Alexa Fluor 555 Goat α-Mouse Invitrogen, #A21422 https://www.thermofisher.com/antibody/product/Goat-anti-Mouse-IgG-H-L-Cross-Adsorbed-Secondary-Antibody-Polyclonal/A-21422
Alexa Fluor 555 Donkey α-Rabbit Invitrogen, #A31572 https://www.thermofisher.com/antibody/product/Donkey-anti-Rabbit-IgG-H-L-Highly-Cross-Adsorbed-Secondary-Antibody-Polyclonal/A-31572
Biotinylated Goat α-Mouse Vector Lab,#BA-9200 https://vectorlabs.com/products/biotinylated-goat-anti-mouse-igg/

Eukaryotic cell lines

Policy information about cell lines and Sex and Gender in Research

Cell line source(s)	Huh7 cell line was from the Sarnow lab (Stanford), and Hep3B cells were purchased from ATCC.
Authentication	Validation was performed by STR profiling using ATCC cell authentication service (ASN-0002-2022).
Mycoplasma contamination	Routine testing for mycoplasma was conducted by MycoAlertTM Mycoplasma Detection Kits (Lonza, 75870-454). All cells tested negative for mycoplasma.
Commonly misidentified lines (See ICLAC register)	No commonly misidentified cell lines were used.

Animals and other research organisms

Policy information about studies involving animals; ARRIVE guidelines recommended for reporting animal research, and Sex and Gender in Research

Laboratory animals	All studies were approved by the Stanford APLAC or Palo Alto VA, and were strictly following the ARRIVE Guidelines.<br>Wild type C57BL/6J (WT) 8- to 10-week-old male mice were purchased from the Jackson Laboratory. Ragefl/fl mice on a C57B6 background were gifted by Dr. B. Arnold from German Cancer Research Center, Heidelberg, Germany. RageHepKO mice were generated by crossing Ragefl/fl mice with Albumin-cre mice (the Jackson Laboratory) for several generations. Eight- to 10-week-old male Ragefl/fl mice and RageHepKO mice were used. To generate hepatocyte transgenic AGER1 mice, WT mice were injected with adeno-associated virus 8 (AAV8)-control green fluorescent protein (AAV8-control) or AAV8-thyroxine-binding globulin-AGER1 recombinase (AAV8-AGER1) (5x10e11 genome copies, Vector BioLabs) at week 6th of feeding.<br>To knockdown TNS1, mice were injected with CRISPR/Cas9-based vector linking two sgRNAs targeting TNS1 exon 1 and exon 7 (pX333-TNS1, 50 µg) or negative control vector (empty pX333, 50 µg), by hydrodynamic tail vein injection at the beginning of the 8th week of chow or HiAD feeding at the same time as with hMet, s45y-mutant- β-catenin, and SB<br>All mice used were on the same background and kept in the same facility. Mice were maintained at macroenvironmental temperature and humidity ranges of 17.8 to 26.1 °C and 30% to 70%. Mice were housed in standard cages with 12:12 hour light/dark cycles and ad libitum access to water and food unless otherwise indicated.
Wild animals	This study did not involve wild animals.
Reporting on sex	Only male mice were used.
Field-collected samples	This study did not involve samples collected in the field.
Ethics oversight	All animal experiments were conducted according to the experimental procedures approved by the Institutional Animal Care and Use Committee at Stanford University and Palo Alto VA. (APLAC #33374)

Note that full information on the approval of the study protocol must also be provided in the manuscript.
